# Virtual biopsy using MRI radiomics for prediction of BRAF status in melanoma brain metastasis

**DOI:** 10.1038/s41598-020-63821-y

**Published:** 2020-04-20

**Authors:** Ben Shofty, Moran Artzi, Shai Shtrozberg, Claudia Fanizzi, Francesco DiMeco, Oz Haim, Shira Peleg Hason, Zvi Ram, Dafna Ben Bashat, Rachel Grossman

**Affiliations:** 1 Department of Neurosurgery, Tel Aviv Medical Center, and the Sackler Faculty of Medicine, Tel-Aviv University, Tel-Aviv, Israel; 2 Sagol Brain Institute, Tel Aviv Medical Center, and the Sackler Faculty of Medicine and Sagol School of Neuroscience, Tel Aviv University, Tel-Aviv, Israel; 3 Department of Neurosurgery, Fondazione IRCCS Istituto Neurologico C. Besta, Milan, Italy; 4 Division of Oncology, Tel Aviv Medical Center, Tel Aviv, Israel and the Sackler Faculty of Medicine, Tel-Aviv University, Tel-Aviv, Israel

**Keywords:** Cancer in the nervous system, Cancer imaging

## Abstract

Brain metastases are common in patients with advanced melanoma and constitute a major cause of morbidity and mortality. Between 40% and 60% of melanomas harbor BRAF mutations. Selective BRAF inhibitor therapy has yielded improvement in clinical outcome; however, genetic discordance between the primary lesion and the metastatic tumor has been shown to occur. Currently, the only way to characterize the genetic landscape of a brain metastasis is by tissue sampling, which carries risks and potential complications. The aim of this study was to investigate the use of radiomics analysis for non-invasive identification of BRAF mutation in patients with melanoma brain metastases, based on conventional magnetic resonance imaging (MRI) data. We applied a machine-learning method, based on MRI radiomics features for noninvasive characterization of the BRAF status of brain metastases from melanoma (BMM) and applied it to BMM patients from two tertiary neuro-oncological centers. All patients underwent surgical resection for BMM, and their BRAF mutation status was determined as part of their oncological work-up. Their routine preoperative MRI study was used for radiomics-based analysis in which 195 features were extracted and classified according to their BRAF status via a support vector machine. The BRAF status of 53 study patients, with 54 brain metastases (25 positive, 29 negative for BRAF mutation) was predicted with mean accuracy = 0.79 ± 0.13, mean precision = 0.77 ± 0.14, mean sensitivity = 0.72 ± 0.20, mean specificity = 0.83 ± 0.11 and with a 0.78 area under the receiver operating characteristic curve for positive BRAF mutation prediction. Radiomics-based noninvasive genetic characterization is feasible and should be further verified using large prospective cohorts.

## Introduction

Melanoma is the third most common cutaneous tumor after basal cell carcinoma and squamous cell carcinoma. Melanomas account for up to 1.6% of newly diagnosed malignancies in the developed world^1^. The worldwide incidence of malignant melanoma is rising, with approximately 290,000 new cases diagnosed per year, resulting in up to 60,000 mortalities^2^. Systemic melanoma carries a high risk of central nervous system (CNS) spread^3^. Up to 75% of stage IV patients with systemic melanoma eventually develop CNS metastases, which account for up to 50% of melanoma-related mortalities^4,5^. The risk of brain metastases from melanoma (BMM) rises with disease duration. While 20–30% of patients will develop BMM in one year, 30–40% will develop BMM in 3 years^6^.

Identification of BRAF activating mutation as a key oncogene in approximately half of all melanomas has dramatically changed the current treatment strategy for metastatic melanoma^7–10^. BRAF mutation has been assumed to be an early evolutionary event in tumor maturation, and play a central role in melanoma pathogenesis^11^. At the cellular level, BRAF mutations drive oncogenic behavior of melanoma cells, leading to unrestricted cell growth, increased cell survival, and local invasion through activation of the mitogen-activated protein kinase (MAPK) pathway^11^. Following identification of this mutation, combination therapy with BRAF and MEK inhibitors have improved patient outcomes dramatically, with the median overall survival (OS) of patients with metastatic melanoma increasing from approximately 9 months before the introduction of these treatments to over 2 years in 2019^12,13^.

Recent studies have shown that primary tumors and brain metastases do not always share the same mutation status^14^. Discrepancies of BRAF mutation status between the primary tumor and the distant metastases reportedly range from 18% to 26%, and patients with a BRAF negative primary melanoma may still manifest BRAF positive BMM and vice versa^15^. This information is crucial for appropriate management when considering non-surgical treatment for a brain metastasis. In addition, prolonged use of BRAF inhibitors can induce BRAF inhibitor resistance and secondary skin tumors^16^, further emphasizing the need for mutation identification in each metastasis, instead of empiric treatment. Currently, the BRAF mutation status of a metastasis cannot be determined without invasively obtaining tissue samples during surgery, which is associated with morbidity, hospitalizations, and is prone to sampling errors.

Radiomics is a field of medical study that aims to achieve tissue characterization using extraction of large numbers of quantitative features from imaging studies. Radiomics analysis, based on magnetic resonance imaging (MRI) data, may be used to characterize pathologies beyond what can be observed by the radiologist’s “naked” eye. By means of this approach, standard imaging studies are converted into high-dimension quantitative data, potentially better reflecting the underlying pathology and its molecular characteristics^17^. We hypothesized that radiomics may provide a noninvasive means of improving decision-making in melanoma neuro-oncologic therapeutic management, thereby possibly sparing the need for invasive procedures and their associated morbidity. In this work, we applied radiomics analysis on conventional MRI-derived data in order to predict the BRAF mutation status in patients with BMM.

## Methods

### Data acquisition and ethical approval

Clinical and imaging data were collected from two tertiary neurosurgical referral centers (Tel Aviv Sourasky Medical Center, Tel-Aviv, Israel [40 patients] and Fondazione IRCCS Istituto Neurologico C. Besta, Milan, Italy [13 patients]). Table 1 lists the demographic and BRAF characteristics of the study patients. The study protocol was approved by the institutional review boards (IRBs) in both centers (IRB approval numbers 0200–10, and 53/2018, from the Fondazione IRCCS Istituto Neurologico C. Besta IRB, and the Tel Aviv Sourasky Medical Center IRB respectively). No informed consent was required by the IRB at either center for this retrospective study which utilized anonymous data. All procedures were carried out in accordance with relevant guidelines and regulations.

**Table 1:** Patient demographics and BRAF characteristics.

Variable	Total	BRAF positive	BRAF negative	*p*
Number	54	25	29	
Age at surgery, years	60 ± 13.8	55 ± 14	64.4 ± 12.3	0.01
Female/male, n	21/32	9/16	12/17	
Metastasis size, mm^3^	19.62 ± 18.3	21.5 ± 21.2	18 ± 15.6	0.498

Values are mean ± standard deviation.

### Imaging protocol

The analyses were performed on post-contrast 3D T1-weighted MRIs (T1W + c) collected retrospectively from patients’ routine clinical assessments, which had been performed at different sites with different MRI vendors, systems, and acquisition parameters. Specifically, 21 scans were performed on General Electric (GE) systems, 20 on Siemens systems, 13 on a Philips MRI system, 33 on 1.5 Tesla MRI systems, and 21 on 3.0 Tesla MRI systems. The voxel size (mean/median) was 0.9/0.94 * 0.9/0.94, with a slice thickness of 1.6/1.6 mm.

**Figure 1:**
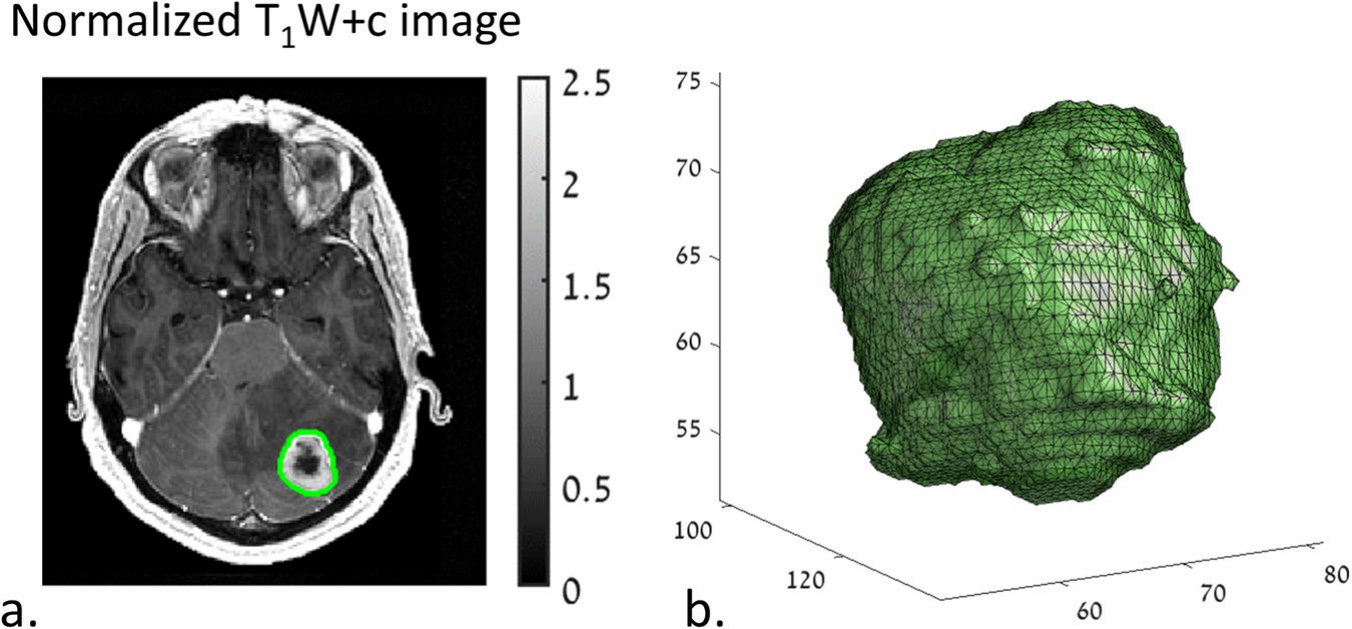
Tumor segmentation. (**a**) Manual delineation of tumor area (green) superimposed on a normalized T1W + c image (normalized relative to normal-appearing white matter). (**b**) 3D view of the extracted lesion.
- Feature extraction and radiomics analysis performed based on the normalized T1W + c image at the entire (3D) lesion area.

Image Analysis. A Matlab (2018a) environment was employed for image analysis which included the following:

- MRI data preprocessing included skull removal and intensity normalization relative to the normal-appearing white matter area, both performed with a statistical parametric mapping segmentation tool (SPM 12).
- Tumor segmentation by a commercial software (AnalyzeDirect 11.0) performed at the slice (2D) level and based on the T1W + c images. The extracted mask was then used to define the 3D target tumor area based on the normalized T1W + c image (Fig. 1).

A total of 195 features were extracted for each patient including:

- Clinical and demographic data (age and gender).
- Sixteen location features calculated as the percent of the segmented (3D) lesion area in each of the 16 brain regions (the left and right frontal, parietal, temporal, occipital, limbic, sublobar lobes, cerebellum, and brainstem, defined according to the Talairach space anatomy template^18^).
- Nine morphological features parameters calculated by Matlab regionprops3 function, including volume, EquivDiameter extent, principal axis length (for the x, y, and z axes), ConvexVolume, solidity, and SurfaceArea.
- Twelve first-order statistical features extracted by analyses using Matlab, including the mean, median, standard deviation, variance, minimum, maximum, percentile (5, 25, 75, and 95), kurtosis, and skewness. And on one-hundred and fifty-six second-order statistical features (gray-level co-occurrence matrix, n = 156) calculated with the Matlab cooc3d function^19^ and based on Haralick *et al*.^20^, including energy, entropy, correlation, contrast, variance, sumMean, inertia, cluster shade, cluster tendency, homogeneity, maxProbability, and inverse variance. Those 12 parameters were calculated with a distance of 8 between voxels (based on experimental results) and along 13 offset, 4 standard 2D directions, with offset (angle between the pixel of interest and its neighbor) = 0°, 45°, 90°, 135°, and an additional 9 directions to extract the 3D information.
- For dimensional reduction, the Matlab kstest, ranksum, ttest2, and pcacov functions were applied to the 195 features in order to reduce the number of random variables under consideration and to improve the classification results. All features were standardized before classification according to:

$${X}_{SDi}=[{X}_{i}-\hat{X}]/\sigma \hat{X}$$

where *x*_*i*_ = value of the individual subject for a given feature, and $\hat{X}\,$and $\sigma \hat{X}$ = mean and standard deviation value of the entire group for a given parameter.

### Statistical analyses

The one-sample Kolmogorov-Smirnov test was used to test the distribution of each feature. Significant differences (*p* < 0.05) between groups (tumoral versus non-tumoral components) were tested using the Mann-Whitney U-test or t-test (depending upon data distribution). Next, principal component analysis (PCA) was applied only on features that were significantly different (*p* < 0.05) between groups.

### Classification and evaluation of the results

Following dimensional reduction and classification of the BRAF mutation status, several support vector machine (SVM) classifier types were tested, including linear, quadratic, cubic, fine gaussian, medium gaussian, and coarse gaussian. This algorithm was chosen after having been shown to produce better results in various brain tumors classification tasks compared to other conventional machine-learning classifiers^21–23^. The results were evaluated by means of a 5-fold cross-validation scheme of randomly splitting the data into training and validation sets (42 and 11 patients, respectively), while maintaining the correct patient representation in each of the five data sets. Precision, sensitivity, specificity, accuracy, and receiver operating characteristics (ROC) curves were calculated for each data set (Table 2).

**Table 2:** The 5-fold validation results.

Data set	BRAF positive (n = 25)			BRAF negative (n = 29)			Model accuracy
	Precision	Sensitivity	Specificity	Precision	Sensitivity	Specificity	
1	1.00	1.00	1.00	1.00	1.00	1.00	1.00
2	0.80	0.80	0.83	0.83	0.83	0.80	0.82
3	0.60	0.60	0.67	0.67	0.67	0.60	0.64
4	0.67	0.40	0.83	0.63	0.83	0.40	0.64
5	0.80	0.80	0.80	0.80	0.80	0.80	0.80
Mean ± standard deviation	0.77 ± 0.14	0.72 ± 0.20	0.83 ± 0.11	0.79 ± 0.13	0.83 ± 0.11	0.72 ± 0.20	0.79 ± 0.13

## Results

### Clinical characteristics

We conducted a retrospective analysis of data obtained from 53 melanoma patients with CNS involvement who underwent resection of their BMM tumors and for whom the BRAF mutation status was available. We retrieved 54 post-contrast 3D T1-weighted MRI scans for those 53 patients (one patient underwent resection of two separate metastases and therefore analysis was carried independently for each metastasis). The patients’ demographic and BRAF characteristics are presented in Table 1.

**Figure 2:**
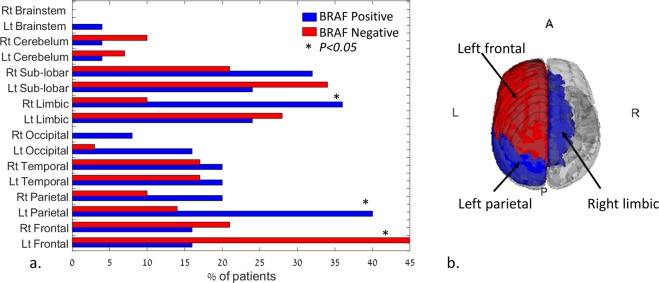
(**a**.) Boxplot of age differences detected between patients with positive (59.8 ± 14.5 years old) and negative (69.2 ± 11.0 years old) BRAF mutation. *= significant difference, *p* = 0.0092. (**b**.) Spider plot of significant difference (*p* < 0.05) detected for the second-order statistical features (significant mean group differences across the different offsets).

**Figure 3:**
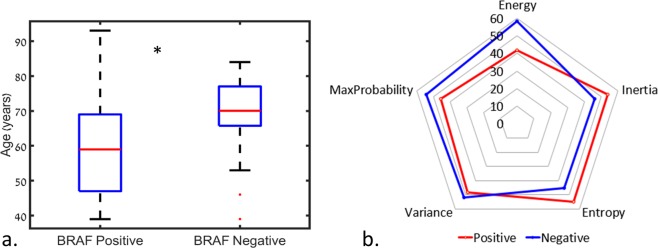
(**a**) Bar plot of tumor location differences detected between patients with positive and negative BRAF mutations. Significant differences (*p < 0.005) were detected for the left frontal, right limbic, and left parietal area. (**b**) 3D visualization of the location with significant differences between groups (red = negative BRAF mutation, blue = positive BRAF mutation).

Twenty-five tumors were positive and 29 were negative for BRAF mutation. Patients with a BRAF mutation were younger (59.8 ± 14.5 years) than those harboring non-mutated tumors (69.2 ± 11.0 years, *p* = 0.0092, Fig. 2a). Fifty out of 195 features significantly (*p* < 0.05) differentiated between tumors with BRAF mutations and those without BRAF mutations. Of those features, 46 were second-order statistical features (Fig. 2b) and three were anatomical, with BRAF-positivef lesions more common in the right limbic and left parietal regions, and BRAF-negative lesions more common in the left frontal area (Fig. 3, a + b). Second-order statistical differences were detected for the features of energy, max probability, variance, entropy, and inertia (Fig. 2b), with the top three features being energy at 90 degrees (2D direction), MaxProbability at 45/135 degrees, and MaxProbability at 135/135 degrees (3D directions), indicating higher tumor signal heterogeneity for patients with negative BRAF mutations. Following the application of PCA on the 50 significant features, nine components were found to explain 95% of the variance and they were subsequently used for classification.

### Classification results

**Figure 4:**
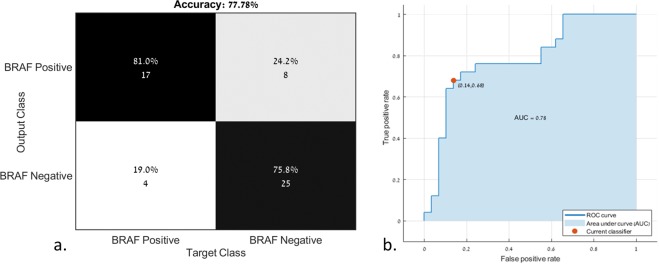
(**a**.) Confusion matrix and (**b**) receiver operating characteristics curve (ROC) for the classification of positive and negative BRAF mutations.

Classification between tumors with and without BRAF mutations was based on the nine principal components extracted from the 50 significant radiomics features and with the use of six types of SVM classifiers. The best classification results were obtained using the linear SVM classifier, with mean accuracy = 0.79 ± 0.13, mean precision = 0.77 ± 0.14, mean sensitivity = 0.72 ± 0.20, mean specificity = 0.83 ± 0.11 for BRAF-positive, and mean precision = 0.79 ± 0.13, mean sensitivity = 0.83 ± 0.11, and mean specificity = 0.72 ± 0.20 for BRAF-negative. The area under the curve (AUC) of the ROC curve was 0.78 for the prediction of a positive BRAF mutation. The classification results for each of the five iterations are given in Table 2. Figure 4 illustrates the confusion matrix of the classification results (Fig. 4a) and the ROC curve for the classification of positive and negative BRAF mutations (Fig. 4b).

## Discussion

The results of this study demonstrate a proof of concept for virtual biopsy using radiomics analysis for the noninvasive diagnosis of BRAF mutation status in BMM. The MRI studies of 54 BMM with known BRAF status were designated to undergo radiomics analysis for the extraction of selected features, and submitted to machine learning in order to classify them according to their mutation status. Despite the limited number of patients, our preliminary results from a linear SVM classifier demonstrated 78% accuracy.

The application of radiomics and machine learning for tumor classification in modern oncology and neuro-oncology is rapidly gaining momentum, and its gradual transformation from an experimental tool to an auxiliary clinical tool can be expected in the not-too-distant future. In parallel to the development of artificial intelligence, neural networks, and deep learning-based classification models, novel radiological biomarkers are being identified and introduced into various aspects of the clinical setting. Trebeschi *et al*. recently demonstrated that standard of care body imaging can be used via radiomics-based analysis to generate a noninvasive biomarker for the response to immunotherapy in systemic malignancies. They reported that this biomarker predicted response to anti-PD1 therapy with an AUC of 0.83 for patients with non-small-cell lung carcinoma, and that the application of an integrated model predicted a survival advantage of 24% following immunotherapy using their classifier in a subgroup of those patients^24^.

A noninvasive, radiomics-based approach would be highly desirable in the brain, where invasive tissue sampling is sometimes associated with surgical complications. Three main areas that may especially benefit from this approach have been recently studied: imaging-based pathological diagnosis (primary vs secondary, low- vs high-grade, and origin of metastasis), identification of treatable mutations or of mutations that influence therapeutic decision-making, and prognostic classification of patients^25–29^. Kniep *et al*. recently classified the five most common BMM according to the primary tumor based on heterogenous clinical MRI scans obtained routinely during the clinical workup. In their study, random forest machine-learning algorithms yielded an AUC ranging from 0.64 to 0.82, depending upon tumor type^30^. Despite this success in identifying the tumor type, no attempt was made to further characterize the genetic landscape of a specific melanoma metastasis. With a similar rationale as that of our current study, the utilization of radiomics for the detection of BRAF and CTNNB1 mutations in craniopharyngioma patients was successfully demonstrated by Chen *et al*., who achieved a level of accuracy of 0.93 in the detection of mutation status, and demonstrated the promising potential of radiomics for those mutations^31^. Della Seta *et al*. used three-dimensional quantitative tumor enhancement, an approach similar to our methodology, in order to stratify patients harboring BMM into prognostic groups^32^. These studies and others that were recently published focused mainly on noninvasive diagnoses, and not on genetic landscape characterization^26,29^. Classification of treatable mutations, however, remains poorly investigated.

Bordia *et al*. used traditional radiological and genetic characteristics in order to assess the prognosis of patients with BMM. Aside from the number of metastases, they reported that BRAF mutation status and concomitant BRAF inhibitor treatment was the most significant prognostic factor. No correlation was found between traditional radiological features and BRAF status, further emphasizing the need for novel radiological biomarkers for this mutation^33^. Since the use of BRAF inhibitors have dramatically improved the prognosis of advanced-stage melanoma and allow for long-term control in some cases, it is critical to assess the mutation status of the tumor^12,34–36^. This especially holds true for patients with non-resectable BMM who are candidates for stereotactic radiosurgery. For these individuals, the addition of BRAF targeted therapy to the radiation treatment was shown to have significant benefit, thus making the potential establishment of a noninvasive diagnostic method even more valuable^37^. In addition, recent evidence has demonstrated a discrepancy between the genetics of the primary tumor and its CNS metastasis in 13.4% of cases and in 7% of lesions in patients with polymetastatic disease^38,39^. Thus, the need for the development of a noninvasive tool that allows sequential evaluation of the genetic status of multiple metastases is clear.

Our tool, which is presented herein, represents a proof of concept for the noninvasive identification of the genetic status of BMM. Using our classifier, it was possible to achieve an AUC of 0.78 despite the limited number of sample subjects. The positive predictive value and the negative predictive value in our study were 81% and 75.8%, respectively. Although these results are lower than the traditional histology-based results^40^, they may still be useful in polymetastatic or fragile patients who are not optimal surgical candidates. Doing so may spare these patients from the need to undergo invasive brain tissue sampling, yet will still enable them to receive targeted biological therapy.

The main limitation of our study stems from the relatively small number of patients. However, given the fact that this work is a collaboration between two neuro-oncological centers with a relatively large neurosurgical volume load, the low power represents a real-world problem of tissue rarity and limited availability. This is especially true in an era when alternatives to surgical resection such as radiosurgery and targeted biological therapy are becoming more popular. For this exact reason we believe that noninvasive mutation characterization will be even more valuable in the future.

## Conclusion

Noninvasive classification of BRAF status in BMM based on MRI findings is feasible and may enable treatment optimization in patients unfit for surgery. It may also aid in choosing patients for neoadjuvant targeted therapy. This approach allows for sequential estimation of genetic mutations by means of routine clinical imaging. Given that radiomics and other advanced imaging-based approaches may be easily compiled and shared online for public use, the need for tissue databanks, online imaging repositories, and multicenter collaborative studies is that much more pressing. Radiomics is a promising technology that may lead to noninvasive characterization of the genetic landscape of brain metastasis, and it warrants further studies on larger cohorts.

## References

[1] Ferlay J (2015). Cancer incidence and mortality worldwide: Sources, methods and major patterns in GLOBOCAN 2012: Globocan 2012. Int. J. Cancer.

[2] Bray F (2018). Global cancer statistics 2018: GLOBOCAN estimates of incidence and mortality worldwide for 36 cancers in 185 countries. CA Cancer J Clin.

[3] Cohen JV (2016). Melanoma central nervous system metastases: current approaches, challenges, and opportunities. Pigment Cell Melanoma Res..

[4] Hannan EJ (2017). The significance of BRAF V600E mutation status discordance between primary cutaneous melanoma and brain metastases: The implications for BRAF inhibitor therapy. Medicine.

[5] Chamberlain MC (2010). Brain metastases: a medical neuro-oncology perspective. Expert Rev Neurother.

[6] Chiarion-Sileni V (2011). Central nervous system failure in melanoma patients: results of a randomised, multicentre phase 3 study of temozolomide- and dacarbazine- based regimens. Br. J. Cancer.

[7] Davies H (2002). Mutations of the BRAF gene in human cancer. Nature.

[8] Flaherty KT (2012). Combined BRAF and MEK inhibition in melanoma with BRAF V600 mutations. N. Engl. J. Med..

[9] Larkin J (2014). Combined vemurafenib and cobimetinib in BRAF-mutated melanoma. N. Engl. J. Med..

[10] Robert C (2015). Improved overall survival in melanoma with combined dabrafenib and trametinib. N. Engl. J. Med..

[11] Dhomen N (2009). Oncogenic Braf Induces Melanocyte Senescence and Melanoma in Mice. Cancer Cell.

[12] Luke JJ, Flaherty KT, Ribas A, Long GV (2017). Targeted agents and immunotherapies: optimizing outcomes in melanoma. Nat Rev Clin Oncol.

[13] Robert, C. *et al*. Five-Year Outcomes with Dabrafenib plus Trametinib in Metastatic Melanoma. *N. Engl. J. Med*. 10.1056/NEJMoa1904059 (2019). 10.1056/NEJMoa1904059 31166680

[14] Brastianos PK (2015). Genomic Characterization of Brain Metastases Reveals Branched Evolution and Potential Therapeutic Targets. Cancer Discov.

[15] Heinzerling L (2013). Mutation landscape in melanoma patients clinical implications of heterogeneity of BRAF mutations. Br J Cancer.

[16] Saroufim M (2014). Comparing BRAF mutation status in matched primary and metastatic cutaneous melanomas: Implications on optimized targeted therapy. Experimental and Molecular Pathology.

[17] Gillies RJ, Kinahan PE, Hricak H (2016). Radiomics: Images Are More than Pictures, They Are Data. Radiology.

[18] Lancaster JL (2000). Automated Talairach atlas labels for functional brain mapping. Hum Brain Mapp.

[19] Philips, C. & Li, D. cooc3d. (2008).

[20] Haralick R, Shanmugam K (1976). Textural features for image classification. IEEE Transactions on systems, man, and cybernetics. IEEE Transactions on systems, man, and cybernetics.

[21] Artzi M, Bressler I, Ben Bashat D (2019). Differentiation between glioblastoma, brain metastasis and subtypes using radiomics analysis. J Magn Reson Imaging.

[22] Blumenthal DT (2017). Classification of High-Grade Glioma into Tumor and Nontumor Components Using Support Vector Machine. AJNR Am J Neuroradiol.

[23] Artzi M (2018). Differentiation between vasogenic edema and infiltrative tumor in patients with high-grade gliomas using texture patch-based analysis: Texture Patch-Based Analysis in HGG. J. Magn. Reson. Imaging.

[24] Trebeschi S (2019). Predicting response to cancer immunotherapy using noninvasive radiomic biomarkers. Annals of Oncology.

[25] Aerts HJWL (2014). Decoding tumour phenotype by noninvasive imaging using a quantitative radiomics approach. Nat Commun.

[26] Shofty B (2018). MRI radiomics analysis of molecular alterations in low-grade gliomas. Int J CARS.

[27] Kickingereder P, Andronesi O (2018). Radiomics, Metabolic, and Molecular MRI for Brain Tumors. Semin Neurol.

[28] Kickingereder P (2016). Radiomic Profiling of Glioblastoma: Identifying an Imaging Predictor of Patient Survival with Improved Performance over Established Clinical and Radiologic Risk Models. Radiology.

[29] Zhou H (2017). MRI features predict survival and molecular markers in diffuse lower-grade gliomas. Neuro Oncol.

[30] Kniep HC (2019). Radiomics of Brain MRI: Utility in Prediction of Metastatic Tumor Type. Radiology.

[31] Chen X (2019). Noninvasive molecular diagnosis of craniopharyngioma with MRI-based radiomics approach. BMC Neurol.

[32] Della Seta M (2019). A 3D quantitative imaging biomarker in pre-treatment MRI predicts overall survival after stereotactic radiation therapy of patients with a singular brain metastasis. Acta Radiologica.

[33] Bordia R (2017). Melanoma brain metastases: correlation of imaging features with genomic markers and patient survival. J Neurooncol.

[34] Forschner A (2017). Improvement of overall survival in stage IV melanoma patients during 2011–2014: analysis of real-world data in 441 patients of the German Central Malignant Melanoma Registry (CMMR). J Cancer Res Clin Oncol.

[35] Sloot S (2018). Improved survival of patients with melanoma brain metastases in the era of targeted BRAF and immune checkpoint therapies: Survival from Melanoma Brain Metastases. Cancer.

[36] Maxwell R (2017). *BRAF* ‐V600 mutational status affects recurrence patterns of melanoma brain metastasis. International Journal of Cancer.

[37] Hadi, I. *et al*. Stereotactic radiosurgery combined with targeted/ immunotherapy in patients with melanoma brain metastasis. *Radiation Oncology* **15**, (2020). 10.1186/s13014-020-1485-8 PMC7023694 32059731

[38] Valachis A, Ullenhag GJ (2017). Discrepancy in BRAF status among patients with metastatic malignant melanoma: A meta-analysis. European Journal of Cancer.

[39] Mesbah Ardakani N (2017). Clinical and therapeutic implications of *BRAF* mutation heterogeneity in metastatic melanoma. Pigment Cell Melanoma Res..

[40] Long GV (2013). Immunohistochemistry is highly sensitive and specific for the detection of V600E BRAF mutation in melanoma. Am. J. Surg. Pathol..

